# Is there an independent association between metabolic syndrome and smoking in Iranian adults? Results of a large multicenter national survey

**DOI:** 10.22088/cjim.12.3.327

**Published:** 2021-04

**Authors:** Alipasha Meysamie, Reza Ghalehtaki, Saeed Ghodsi, Mehrnaz Mohebi, Shirin Ghalehtaki, Fereshteh Salarvand, Zahra Hosseini, Seyed-Ali Sadre-Bafghi

**Affiliations:** 1 Department of Preventive and Community Medicine, Tehran University of Medical Sciences, Tehran, Iran; 2 Radiation Oncology Research Center (RORC), Cancer Institute, Tehran University of Medical Sciences, Tehran, Iran; 3 Cardiovascular Research center, Department of Cardiology, Tehran Heart Center, Tehran University of Medical Sciences, Tehran, Iran; 4 Department of Obstetrics and Gynecology, Shahid Beheshti University of Medical Sciences, Tehran, Iran; 5 Department of Dermatology, Razi Hospital, Tehran University of Medical Sciences, Tehran, Iran

**Keywords:** Metabolic syndrome, Smoking, Adult, Complex samples, Central obesity, Prevalence, Risk factor, Iran

## Abstract

**Background:**

Theoretically, smoking status should be associated with metabolic syndrome. This relationship has not been studied in Iranian population so far. This study aimed to explore the association among cigarette smoking, metabolic syndrome (MetS) and its domains in a nationally representative sample of Iranians aged 25-64.

**Methods:**

Information of participants regarding demographic data and smoking habits gathered through WHO STEPS questionnaires in the frame of fourth national surveillance of the risk factors of non-communicable diseases in 2011 across the country. The fasting plasma glucose, triglyceride level, high-density lipoprotein cholesterol (HDL-C) level, blood pressure, and anthropometric indices in each patient were measured. Data of 4000 subjects were analyzed with complex sample survey method. The prevalence of metabolic syndrome was assessed according to two definitions: International Diabetes Federation (IDF) and Iranian definition.

**Results:**

Herein, 38.4% of smoker participants and 36.6% of non-smokers met the IDF criteria for MetS (P=0.67). Similarly, 31.1% of smokers and 34.1% of non-smokers had MetS according to Iranian-IDF (P=0.427). Only in univariate analysis, using IDF criteria female smokers had lower prevalence of MetS than non-smokers (13.9% vs. 36.5%, P=0.01). Multivariate analysis determined the following odds ratios for the association of smoking with MetS defined by IDF and Iranian-IDF criteria, respectively: OR= 0.89 (0.53-1.47), P=0.638 and OR= 0.97 (0.59-1.58), P=0.901.

**Conclusion:**

There was no significant association between smoking and MetS overall and among men. However, smoking was associated with lower prevalence of MetS among women.

Smoking represents as a major modifiable risk factor of cardiovascular disease (CVD) and diabetes mellitus (DM). Several mechanisms have been suggested for corresponding pathogenesis. Atherosclerosis is the main pathway through which a variety of substances and particles of tobacco (esp. nicotine) contribute to progression of subsequent CVD and burden of coronary events (1). Disorders of lipid profile, production of oxidized LDL (low density lipoprotein) due to excessive free radicals, marked activation of sympathetic nervous system, pro-thrombotic states triggered via inhibition of t-PA (tissue-plasminogen activator) release from the endothelial wall accompanied by increased platelet aggregation, impaired prostacyclin synthesis, and over-expression of tissue factor have been explained as modulators of tobacco effects.

Reduced capacity of vasodilation and decreased reserve of coronary flow will occur in part due to low nitric oxide generation secondary to endothelial damage (2–7). Although not established but it is thought that nicotine and carbon monoxide, elevated CRP (C-reactive protein) and fibrinogen levels may be also involved in atherosclerosis and associated inflammatory cascades (1,8). Cigarette smoking has been investigated as a risk factor in developing insulin resistance which in turn, may promote diabetes mellitus (9).

Metabolic syndrome (MetS) is the constellation of some metabolic disorders, which has been extensively used as a traditional concept. Insulin resistance has been proposed as the cornerstone of metabolic syndrome denoting how the construct is resulted (10), however, critical arguments have remained within the role of MetS as a unique entity. In other words, it is unclear whether the treatment of individuals with metabolic syndrome improves the clinical outcome as compared with the treatment of MetS components, separately (11). Furthermore, different diagnostic criteria and variety of phenotypes have been suggested (11,12). The relationship between smoking and MetS is influenced by the above-mentioned heterogeneity of the criteria and multiple factors including the components of metabolic syndrome. To date, controversy exists about the presence of a positive association between smoking and MetS, its strength, modifiers, and clinical implications. There are more studies reporting a greater risk of MetS for smokers (13,14) than those who found an inverse association (15). Nevertheless, frequent surveys have demonstrated either no significant association (16) or a gender-specific difference in the relation of MetS and smoking (14,17). Thus, we aimed to evaluate this relationship in a large population of Iranian adults.

## Methods

A cross-sectional study was conducted based on data of national “Surveillance of Risk Factors of Non-Communicable Diseases” in 2011 (SuRFNCD-2011). In brief, the survey had been executed with a randomized clustered sampling scheme using stratification models to achieve a nationally representative sample. Specific questionnaires were used which had been designed according to the STEPS (Stepwise approach to Surveillance) (The Word Health Organization STEPwise approach to Surveillance of non-communicable diseases (STEPS) instrument developed by WHO. It was a standardized but flexible framework consisted of 6 major domains including demographic data, nutritional status, physical activity, tobacco use, history of diabetes mellitus, and hypertension. Further details about the survey have been explained elsewhere (18). In addition, extended information and supplements are available on database of the Ministry of Health and medical education of Iran (ncdinfobase.ir).

In the current study, we have excluded the pregnant women due to their different metabolic profile as well as varied definitions of metabolic syndrome, subjects with missing data regarding demographics, smoking or metabolic profile data and outliers. Four-thousand participants aged 25-64 years old (the age range of main working population) remained ultimately. To detect the cases of metabolic syndrome, two types of classification criteria were applied. International Diabetes Federation (IDF) has described the metabolic syndrome with the presence of abdominal obesity as an essential factor (waist circumference ≥ 94 cm for men and ≥ 80 cm for women) plus 2 or more of the following factors: 1) Elevated triglyceride (TG) (≥ 150) or consumption of TG lowering agents; 2) Low HDL-C (HDL levels < 40 mg/dl and < 50 mg/dl in men and women, respectively); 3) hyperglycemia (Fasting plasma glucose ≥ 100) or consumption of insulin and/or oral hypoglycemic agents; 4) High blood pressure (BP) defined as Systolic pressure ≥ 130 or Diastolic pressure ≥ 85 mmHg or consumption of antihypertensive drugs (19). 

We have also applied the Iranian criteria (modified IDF) for MetS which recommended specific cut-points for detection of central obesity. Waist circumference ≥ 90 cm (in both genders) presented the abdominal obesity (20). Cigarette smoking was defined as regular consumption of cigarette at the time of interview. Smoking pack/year was calculated as the number of cigarette packs containing 20 sticks consumed daily multiplied by years since smoking initiation. 

The SurFNCD-2011 had been verified by the national committee of medical ethics and CDC (Center for Disease Control) of Iran. In summary, all participants had given informed consent for data collection by interviews and then for blood sampling. Furthermore, unique identification codes were recruited in documentations to avoid the potential release of personal characteristics. Data analysis was performed using SPSS V 20.0 for windows. An appropriate weighting schedule was recruited to extrapolate the results for national scale. The structure of reference population for weighing was determined according to the reports of the last national census released in 2011 (21). In summary, the population of Iranian residents in 2011 was determined as the reference values. Based on the 2011 national census, 42,287,987 individuals aged 25–64 years lived in Iran. We estimated total weights according to reference populations using a model with three components. Hence, multiple strata for age groups (25–34, 35–44, 45–54, and 55–64), gender (male, female), and residential area (urban versus rural) were scheduled.

Complex sample survey analysis models were applied. Thus, standardized national estimates including MetS prevalence and its domains were calculated. Continuous variables were expressed as mean with 95% confidence interval while categorical variables were expressed as relative frequency (percentage) with 95% confidence interval. The complex sample chi-square test was opted to compare the frequencies of categorical parameters in univariate analysis. We also used t-test for univariate comparison of continuous variables. Complex sample multiple logistic regression analysis helped to assess independent relationship between MetS and smoking by incorporating the sampling weights. The level of statistical significance was considered 0.05.

## Results

Of the total 4000 participants enrolled in the study, 2,373(60.48%) subjects were females and 2,685 (68.84%) living in urban areas (table 1). The mean age of the study population was 38.4 (CI95%: 37.6- 39.21) years. Detailed metabolic and smoking characteristics of the study subjects have been shown in the table 2. The prevalence of current cigarette smoking was 9.6% in total population, 21.4% among men and 1.9% among women. Smoking pattern subtends smoking initiation age, number of daily cigarette sticks smoked and pack/year (number of daily cigarette packs multiplied by years of smoking). 

The prevalence of MetS with Iranian definition was 33.8% (95 % CI: 31.8-35.8) that corresponds to 14.280 million people in national scale. This figure was 31.4% (95% CI: 28.4-34.5) and 35.3% (95% CI: 32.8-38.0) among men and women, respectively. However, by IDF definition the overall prevalence rate of MetS was 36.8% (95% CI: 34.7-38.9) that equals to 15.542 million 25-64 years individuals in national scale. Among the men and women, this rate was 37.8% (95% CI: 34.4-41.3) and 36.1% (95% CI: 33.5-38.7), respectively. Complex sample logistic regression demonstrated that current smoking was not a predictor of metabolic syndrome neither using IDF definition nor by Iranian-IDF criteria (table 3). The associations of current smoking, total cholesterol (TC), LDL, hip girth, and BMI with MetS as the outcome of interest were shown. Among these factors, TC was associated with MetS in an inverse manner (OR=0.89, P=0.017) while BMI appeared to increase the hazard of MetS showing a trend toward significance (OR: 1.17, P=0.08). Both models were adjusted for all ethnic groups of Iranian residents, occupation, and educational level. It is worth noting that age, sex, and area of residence have been implied in weighing model as baseline strata. 

Using Iranian cut-offs, the prevalence of MetS was 34.1%, among non-smokers vs. 31.1% among smokers (P=0.43). These values were 38.4% and 36.6% by IDF definitions (P=0.67). The prevalence of MetS domains based on smoking status was shown in table 5. As shown, there was no significant association between smoking and MetS domains among male smokers. The prevalence of hypertension, hyperglycemia and central obesity (IDF) was significantly lower among female smokers compared to non-smokers. Corresponding values were (17.8% vs 43.4%, P=0.004), (10.8% vs 22.7%, P=0.008), and (35.0% vs 59.9%, P=0.002), respectively. However, there was no significant difference between smokers and non-smokers in total participants.

**Table 1:** Characteristics of the study sample and corresponding national estimates in 2011

		**Unweighted Count**	**Standard Population**	**% of Total Population**
Gender	Men	1,627	16,712,571	39.52
	Women	2,373	25,575,416	60.48
Residential area	Urban	2,685	29,104,663	68.82
	Rural	1,315	13,183,323	31.18
Age Group	25-34	1,128	17,185,017	40.64
	35-44	821	12,051,216	28.50
	45-54	789	8,408,945	19.88
	55-64	1,261	4,642,807	10.98
Total		4000	42,287,987	100.00

**Table 2:** Description of metabolic and smoking profile of the study participants

		***Men***	***Women***	***P-value***	***Total***
Current smoking	Prevalence	21.4% (18.5-24.7)	1.9% (1.2-3.2)	< 0.001	9.6% (8.3-11.1)
	Number (Pop. Est.)^1^	336 (3,571)	28 (494)		364 (4,066,116)
Age of smoking onset (Mean±SD)		21.37 (20.6-22.1)	27.23 (22.2-32.1)	*<0.01*	22.09 (21.1-22.9)
Smoking quantity (number of daily smoked sticks)		16.49 (14.9-18.0)	10.22 (8.1-12.2)	*<0.01*	15.74 (14.3- 17.1)
Smoking Pack/Year		15.60 (12.56-18.65)	6.77 (2.25-11.29)	*<0.01*	14.54 (11.79- 17.30)
HDL		45.43 (44.36-46.49)	44.69 (43.80-45.58)	NS^2^	45.00 (44.3-45.6)
TG		162.73 (149.6-175.8)	155.79 (146.8-164.7)	NS	158.67 (151.1-166.2)
Waist Circumference		84.60 (83.41-85.79)	85.81 (84.92-86.71)	0.06	85.34 (84.62-86.06)
Waist/Hip ratio		0.87 (0.86-0.88)	0.88 (0.87-0.88)	NS	0.88 (0.87-0.88)
BMI		25.02 (24.61-25.42)	25.70 (24.86-26.55)	NS	25.43 (24.90-25.97)
FBS		101.21 (97.1-105.3)	100.76 (98.0-103.4)	NS	100.95 (98.6-103.2)

^1^ National population estimate in thousands, ^2^ Non-significant (P > 0.05),

**Table 3:** Complex samples multivariate logistic regression models regarding association of current smoking and metabolic syndrome with ATP III and Iranian-IDF criteria

Predictor variables	Model 1 (IDF)	P-value	Model 2 (Iranian-IDF)	P-value
**Current smoking**	0.89 (0.53- 1.47)	0.638	0.97 (0.59-1.58)	0.901
**Total Cholesterol (per 30 mg/dl rise)**	0.95 (0.86- 1.05)	0.318	0.89 (0.80-0.98)	0.017
**LDL (per 30 mg/dl increase)**	1.01(0.97-1.06)	0.590	1.02 (0.98- 1.07)	0.284
**Hip girth (per 10 cm increase)**	0.97(0.83- 1.13)	0.721	0.93 (0.79- 1.09)	0.363
**BMI (per 5 units increase)**	1.11(0.94-1.32)	0.232	1.17 (0.98 - 1.41)	0.088

Odds ratio values were expressed in mean ± standard deviation accompanied with 95 % confidence intervals. Both models were adjusted for ethnicity of Iranian residents, occupation, and educational level. Note that age, sex, and area of residence have been implied in weighting process as baseline strata.

**Table 4:** Univariate analysis regarding the frequency of metabolic syndrome based on smoking status

		***Men***	***Women***	***Total***
IRAN criteria	Smoker	32.5% (25.6-40.2)^1^	21.1% (8.3-44.2)	31.1% (29.7-31.9)
	Non-smoker	31.1% (27.8-34.7)	35.6% (33.0-38.3)	34.1% (31.8-36.2)
	Odds Ratio	1.06 (0.73-1.55)	0.48 (0.16-1.43)	0.87 (0.62-1.22)
	P	0.745	0.182	0.427
IDF ^2^ criteria	Smoker	41.7% (33.7-50.3)	13.9% (5.5-30.9)	38.4% (30.9-46.4)
	Non-smoker	36.8% (33.0-40.7)	36.5% (33.9-39.2)	36.6% (34.5-38.8)
	Odds Ratio	1.23 (0.83-1.82)	0.28 (0.10-0.78)	1.07 (0.76-1.52)
	P	0.296	0.010	0.677

^1^Confidence interval 95% of prevalence, ^2^ International Diabetes Federation, The odds ratio (OR) for a current smoker to be affected with metabolic syndrome, Confidence interval (CI) 95% for OR.

**Table 5:** Univariate analysis regarding the frequency of metabolic syndrome domains compared between smokers and non-smokers

***Domains of MetS*** ^1^			***Men***	***Women***	***Total***
Low HDL		Smoker	52.1% (43.8-60.2)	63.9% (39.9-82.6)	53.5% (45.6-61.2)
		Non-smoker	53.9% (49.7-57.9)	53.6% (50.9-56.4)	53.7% (51.4-56.0)
		P	0.703	0.394	0.957
High Triglyceride		Smoker	37.4% (30.5-44.9)	34.4% (15.7-59.7)	37.0% (30.3-44.3)
		Non-smoker	39.7% (35.9-43.6)	38.1% (35.4-40.9)	38.6% (36.5-40.9)
		P	0.592	0.763	0.670
Insulin resistance/diabetes		Smoker	26.6% (20.8-33.4)	10.8% (4.1-15.3)	24.6% (19.3-30.8)
		Non-smoker	23.6% (20.8-26.7)	22.7% (20.7-24.9)	23.0% (21.3-24.8)
		P	0.396	0.008	0.608
Hypertension		Smoker	38.5% (31.3-46.2)	17.8% (8.0-35.2)	35.9% (29.3-42.2)
		Non-smoker	40.1% (36.4-44.0)	43.4% (40.7-46.1)	42.3% (41.1-43.5)
		P	0.712	0.004	0.1
Central Obesity domain	IRAN criteria	Smoker	54.8% (46.6-62.7)	48.9% (25.7-72.6)	54.1% (46.3-61.7)
		Non-smoker	51.0% (47.1-54.9)	54.6% (51.8-57.3)	53.3% (51.1-55.6)
		P	0.420	0.665	0.859
	IDF criteria	Smoker	62.4% (58.8-68.1)	35.0% (25.1-51.9)	59.1% (51.1-66.6)
		Non-smoker	56.2% (54.1-58.1)	59.9% (57.0-62.7)	58.6% (56.2-60.9)
		P	0.048	0.002	0.911

^1^ Metabolic syndrome

## Discussion

Our objective was to investigate the association of smoking and metabolic syndrome in Iranian population, aged 25-64. Since regional and racial varieties may have a substantial impact on the relationship between smoking and metabolic syndrome (22), we could not be certain whether results reported in other parts of the world are true for our population. On the other hand, central obesity, an essential component of metabolic syndrome, has its various definitions in different ethnicities. Accordingly, previous national studies showed that internationally recommended thresholds lack the validity for diagnosing central obesity in Iran (23). This fact led Iranian experts to suggest a novel cut-off (≥90 cm of waist circumference) for Iranian male and female adult population that correctly predicts the risk of cardiovascular events (20).

 This study showed that metabolic syndrome by itself has relatively high prevalence in our population (approx. more than one-third of 25-64-year individuals). This figure even reaches concerning rates in middle-aged population equal to more than half the 55-64-year adults. In our study, the prevalence rate of metabolic syndrome was not statistically different between men and women but when extrapolated to national estimates, women share the bigger fraction of the burden (about 1.5 times the men). This gender pattern has been suggested by previous studies in the developing countries like Turkey and India (24,25) that is opposite to what is seen in developed countries like US, Australia and Ireland that men possess the greater share (26). 

 Multiple studies have shown that smoking might be related to increased risk of metabolic syndrome. These studies have been conducted in the different parts of the world and considered various definitions of metabolic syndrome and threshold for central obesity (17). Some of these reports came from Asian inhabitants of Taiwan and Japan according to ATP III definitions (27–29). Another group of studies were carried out in Europe high-income countries for instance DESIR in France (30), Masulli et al. in Italy (31), Slagter in Netherlands (32), and Tonstad et al. in Norway (33). The major suspects of observed relationship were higher frequency of low high-density lipoproteins (HDL), elevated triglycerides (TG) and greater waist circumference among smokers compared to non-smokers. 

 We did not find an independent association between current smoking and metabolic syndrome for the whole population, which was persistent regarding different criteria (IDF and Iranian-IDF). Furthermore, subgroup analysis in men did not show a considerable association. In spite of men, an inverse association was seen between smoking and metabolic syndrome among women. Indeed, female smokers were significantly less likely to be affected by metabolic syndrome. Although the low number of smoking female subjects and our cross-sectional design of study may limit our interpretation of the relationship but there are some explanations for such finding. Despite earlier mentioned studies, there are some reports with similar findings. In Yang et al.’s study, the same gender paradigm was observed (34). In addition, in another report from our neighboring country Turkey, with largely similar ethnicity and socioeconomic status, the protective role of smoking on metabolic syndrome was suggested in whole population and among women in particular (15). A large Korean study also indicated that the expected dose dependent relationship between smoking and metabolic syndrome is not observed among women despite men (35). In a prospective Turkish study, less prevalence of obesity and insulin resistance among smokers were the major causes of protective effect of smoking on metabolic syndrome (36). Thus, these similarities highlight the importance of geographic differences on the relationship between smoking and metabolic syndrome. In our study, central obesity, hypertension and glucose tolerance seemed to mediate the relationship between smoking and metabolic syndrome among females. Inverse association between smoking and obesity was pointed out by previous studies in our neighboring countries (37–39). The protective effect of smoking on central obesity seems to be independent of insulin resistance and general obesity. Smoking may decrease obesity by diminishing appetite and augmenting metabolism rate. In this regard, a conventional belief explains an inverse association between nicotine as an ingredient of tobacco smoke and obesity. This outcome might be driven via appetite suppressing effect of nicotine, which is a stimulant substance, as well as desensitization of taste buds. However this theory (40). The exclusive effect of smoking against hypertension among women was reported by Primatesta et al. (41) who indicated the protective effect of light smoking against hypertension. Authors believed that this was due to interaction with concomitant alcohol intake and lower body mass index among smokers. In case of lower impaired glucose tolerance and insulin resistance among smokers. Onat et al. (15) achieved similar results among Turkish population. In addition, a Swedish study showed that although smoking may alter beta cell function and has a negative impact on insulin resistance, this phenomenon was not observed among women (42). 

Previous national studies stated that men had greater adjusted 10-year risk of cardiovascular events (according to Framingham and SCORE calculators) than the women, while C-reactive protein level were identical in all age groups (43). Given this fact, the observed link between females who were current smokers and MetS, may refer to etiologies other than underlying inflammation. Moreover, complex samples survey provides more accurate analysis optimized according to the real picture of whole Iranian population groups, sex, age, area of residence, and occupation. It might underscore the effect of analysis and sample selection for the interpretation of discordance between our findings and prior publications. 

A meta-analysis demonstrated that smoking was associated with greater risk of metabolic syndrome in men but no relationship was found in women (17). In contrast with some prior studies (13, 14, 17), we found no association in the whole population as well as in males. Furthermore, the apparently protective effect of smoking in females was not confirmed in multivariate analysis. One hypothesis for the absence of a significant relationship between smoking and MetS might be due to exposure misclassifications. Thus, lack of data about passive smoking, and reporting bias especially in females might have played a role. 

Some authors believe that smoking by itself does not decrease the chance of being affected by metabolic syndrome but the special life style factors like physical inactivity and concurrent alcohol consumption are the protagonists (16,22). Thus, when we control the effect of such life style factors other than ethnicity, we may correctly investigate the association between smoking and metabolic syndrome. In other words, to confidently make a statement toward the relationship between smoking and metabolic syndrome, we need to take the effect of confounding factors including alcohol intake, physical activity, nutritional status, age and socioeconomic status into account.

**Study Limitations and Strengths: **One of the significant limitations of our study was that we could not investigate the frequency of metabolic syndrome in ex-smokers because the number of ex-smokers in our samples was so few and we could not enroll them in the analysis. In addition, we did not gain access to the frequency of other popular types of smoking including occasional water pipe and pipe smoking and their potential link to metabolic abnormalities. Nevertheless, the main advantage of this study was using complex sample survey method to analyze data and extrapolating the conclusion to all 25-64 aged Iranian in 2011.

In conclusion, metabolic syndrome has relatively high prevalence in 25-64 year old Iranian population. Considering ethnicity and Iranian life style in comparison to developed countries or those with different ethnicity and life style, cigarette smoking did not have an independent association with metabolic syndrome. In investigating the association between metabolic syndrome and smoking, various ethnicities and important lifestyle factors should be considered.

**List of Abbreviations**

DM: Diabetes mellitus

CHD: Coronary heart disease

MI: Myocardial Infarction

NCDs: Non-communicable diseases 

CRP: C-reactive protein

MetS: Metabolic syndrome

SuRFNCD: Surveillance of Risk Factors of Non-communicable Diseases

HDL: High-density lipoprotein

STEPS: STEPwise approach to Surveillance

CDC: Center for disease control

BP: Blood pressure

CVD**: **Cardiovascular disease

LDL: Low-density lipoprotein

t-PA : tissue-Plasminogen Activator

NCEP-ATP**: N**ational cholesterol education Program’s Adult Treatment Panel

IDF: International Diabetes Federation

TG: triglyceride

**Declarations**

- Ethics approval and consent to participate

Our study was reviewed and approved by the Ethics Committee, Deputy of Research, Medical school, Tehran University of Medical Sciences.

- Consent for publication

Not applicable

- Availability of data and material

Please contact corresponding author for data requests

## References

[1] Leone A, Landini L (2013). Vascular pathology from smoking: look at the microcirculation! Curr Vasc Pharmacol. Curr Vasc Pharmacol.

[2] Facchini FS, Hollenbeck CB, Jeppesen J, Chen YD, Reaven GM (1992). Insulin resistance and cigarette smoking. Lancet.

[3] Reaven GM (2003). Insulin resistance/compensatory hyperinsulinemia, essential hypertension, and cardiovascular disease. J Clin Endocrinol Metab.

[4] Miller ER, Appel LJ, Jiang L, Risby TH (1997). Association between cigarette smoking and lipid peroxidation in a controlled feeding study. Circulation. Am Heart Assoc.

[5] Cryer PE, Haymond MW, Santiago J V, Shah SD (1976). Norepinephrine and epinephrine release and adrenergic mediation of smoking-associated hemodynamic and metabolic events. N Engl J Med. Mass Medical Soc.

[6] Newby DE, Wright RA, Labinjoh C (1999). Endothelial dysfunction, impaired endogenous fibrinolysis, and cigarette smoking. Circulation.

[7] Barua RS, Ambrose JA, Eales-Reynolds LJ (2001). Dysfunctional endothelial nitric oxide biosynthesis in healthy smokers with impaired endothelium-dependent vasodilatation. Circulation.

[8] Bazzano LA, He J, Muntner P, Vupputuri S, Whelton PK (2003). Relationship between cigarette smoking and novel risk factors for cardiovascular disease in the United States. Ann Intern Med.

[9] Xie X, Liu Q, Wu J, Wakui M (2009). Impact of cigarette smoking in type 2 diabetes development. Acta Pharmacol Sin. Nature.

[10] Grundy SM, Brewer HB, Cleeman JI, Smith SC, Lenfant C (2004). Definition of metabolic syndrome. Circulation. Am Heart Assoc.

[11] Kahn R, Buse J, Ferrannini E (2005). The metabolic syndrome: time for a critical appraisal. Diabetes Care.

[12] Bayturan O, Tuzcu EM, Lavoie A (2010). The metabolic syndrome, its component risk factors, and progression of coronary atherosclerosis. Arch Intern Med.

[13] Kim T, Choi H, Kang J, Kim J (2020). Association between electronic cigarette use and metabolic syndrome in the Korean general population: A nationwide population-based study. Plos One.

[14] Youn JA, Lee YH, Noh MS (2018). Relationship between Smoking Duration and Metabolic Syndrome in Korean Former Smokers. JKSRNT.

[15] Onat A, Ozhan H, Esen AM (2007). Prospective epidemiologic evidence of a "protective" effect of smoking on metabolic syndrome and diabetes among Turkish women-without associated overall health benefit. Atherosclerosis.

[16] Wilsgaard T, Jacobsen BK (2007). Lifestyle factors and incident metabolic syndrome. The Tromsø Study 1979-2001. Diabetes Res Clin Pract.

[17] Sun K, Liu J, Ning G (2012). Active smoking and risk of metabolic syndrome: a meta-analysis of prospective studies. PLoS One.

[18] Hosseini M, Navidi I, Yousefifard M (2014). Serum HDL-C level of Iranian adults: results from sixth national surveillance of risk factors of non-communicable disease. J Diabetes Metab Disord.

[19] Consensus IDF The IDF Consensus worldwide definition of the metabolic syndrome.

[20] Azizi F, Hadaegh F, Khalili D (2010). Appropriate definition of metabolic syndrome among Iranian adults: report of the Iranian National Committee of Obesity. Arch Iran Med.

[21] (2011). Statistical Center of Iran: National Census of Population and Housing of Iran.

[22] Rabaeus M, Salen P, de Lorgeril M (2013). Is it smoking or related lifestyle variables that increase metabolic syndrome risk?. BMC Med.

[23] Esteghamati A, Ashraf H, Rashidi A, Meysamie A (2008). Waist circumference cut-off points for the diagnosis of metabolic syndrome in Iranian adults. Diabetes Res Clin Pract.

[24] Esteghamati A, Khalilzadeh O, Rashidi A (2009). Association between physical activity and metabolic syndrome in Iranian adults: national surveillance of risk factors of noncommunicable diseases (SuRFNCD-2007). Metabolism.

[25] Azizi F, Salehi P, Etemadi A, Zahedi-Asl S (2003). Prevalence of metabolic syndrome in an urban population: Tehran Lipid and Glucose Study. Diabetes Res Clin Pract.

[26] Eckel RH, Alberti KG, Grundy SM, Zimmet PZ (2010). The metabolic syndrome. Lancet.

[27] Wada T, Urashima M, Fukumoto T (2007). Risk of metabolic syndrome persists twenty years after the cessation of smoking. Intern Med.

[28] Ishizaka N, Ishizaka Y, Toda EI (2005). Association between cigarette smoking, metabolic syndrome, and carotid arteriosclerosis in Japanese individuals. Atherosclerosis.

[29] Chen CC, Li TC, Chang PC (2008). Association among cigarette smoking, metabolic syndrome, and its individual components: the metabolic syndrome study in Taiwan. Metabolism.

[30] Geslain-Biquez C, Tichet J, Caradec A (2003). The metabolic syndrome in smokers. The DESIR study. Diabetes Metab.

[31] Masulli M, Riccardi G, Galasso R, Vaccaro O (2006). Relationship between smoking habits and the features of the metabolic syndrome in a non-diabetic population. Nutr Metab Cardiovasc Dis.

[32] Slagter SN, van Vliet-Ostaptchouk JV, Vonk JM (2013). Associations between smoking, components of metabolic syndrome and lipoprotein particle size. BMC Med.

[33] Tonstad S, Svendsen M (2005). Premature coronary heart disease, cigarette smoking, and the metabolic syndrome. Am J Cardiol.

[34] Yang FY, Wahlqvist ML, Lee MS (2008). Body mass index (BMI) as a major factor in the incidence of the metabolic syndrome and its constituents in unaffected Taiwanese from 1998 to 2002. Asia Pac J Clin Nutr.

[35] Oh SW, Yoon YS, Lee ES (2005). Association between cigarette smoking and metabolic syndrome: the Korea National Health and Nutrition Examination Survey. Diabetes Care.

[36] Onat A, Uyarel H, Hergenç G (2007). Determinants and definition of abdominal obesity as related to risk of diabetes, metabolic syndrome and coronary disease in Turkish men: A prospective cohort study. Atherosclerosis.

[37] Al-Nuaim AA, Bamgboye EA, Al-Rubeaan KA, Al-Mazrou Y (1997). Overweight and obesity in Saudi Arabian adult population, role of socio-demographic variables. J Community Health.

[38] Kordy MNS, El-Gamal FM (1995). A Study of Pattern of Body Mass Index (BMI) and prevalence of Obesity in a Saudi Population. Asia Pacific J Public Health.

[39] Erem C, Arslan C, Hacihasanoglu A (2004). Prevalence of obesity and associated risk factors in a Turkish Population (Trabzon City, Turkey). Obes Res.

[40] Nakanishi N, Shiraishi T, Wada M (2005). C-reactive protein concentration is more strongly related to metabolic syndrome in women than in men. Circ J.

[41] Primatesta P, Falaschetti E, Gupta S, Marmot MG, Poulter NR (2001). Association between smoking and blood pressure: evidence from the health survey for England. Hypertension.

[42] Ostgren CJ, Lindblad U, Ranstam J (2000). Hypertension and diabetes project associations between smoking and beta-cell function in a non-hypertensive and non-diabetic population. Skaraborg Hypertension and Diabetes Project. Diabet Med.

[43] Meysamie A, Ghodsi S, Ghalehtaki R (2017). Distributions of high-sensitivity c-reactive protein, total cholesterol-hdl ratio and 10-year cardiovascular risk: National Population-Based Study. Acta Med Iran.

